# Inferring protein domains associated with drug side effects based on drug-target interaction network

**DOI:** 10.1186/1752-0509-7-S6-S18

**Published:** 2013-12-13

**Authors:** Hiroaki Iwata, Sayaka Mizutani, Yasuo Tabei, Masaaki Kotera, Susumu Goto, Yoshihiro Yamanishi

**Affiliations:** 1 Division of System Cohort, Multi-scale Research Center for Medical Science, Medical Institute of Bioregulation, Kyushu University, 3-1-1 Maidashi, Higashi-ku, Fukuoka, Fukuoka 812-8582, Japan; 2 Bioinfromatics Center, Institute for Chemical Research, Kyoto University, Gokasho, Uji, Kyoto 611-0011, Japan; 3 PRESTO, Japan Science and Technology Agency, Kawaguchi, Saitama 332-0012, Japan; 4 Institute for Advanced Study, Kyushu University, 6-10-1, Hakozaki, Higashi-ku, Fukuoka, Fukuoka 812-8581, Japan

## Abstract

**Background:**

Most phenotypic effects of drugs are involved in the interactions between drugs and their target proteins, however, our knowledge about the molecular mechanism of the drug-target interactions is very limited. One of challenging issues in recent pharmaceutical science is to identify the underlying molecular features which govern drug-target interactions.

**Results:**

In this paper, we make a systematic analysis of the correlation between drug side effects and protein domains, which we call "pharmacogenomic features," based on the drug-target interaction network. We detect drug side effects and protein domains that appear jointly in known drug-target interactions, which is made possible by using classifiers with sparse models. It is shown that the inferred pharmacogenomic features can be used for predicting potential drug-target interactions. We also discuss advantages and limitations of the pharmacogenomic features, compared with the chemogenomic features that are the associations between drug chemical substructures and protein domains.

**Conclusion:**

The inferred side effect-domain association network is expected to be useful for estimating common drug side effects for different protein families and characteristic drug side effects for specific protein domains.

## Background

Most phenotypic effects of drugs are involved in the interactions between drugs and their target proteins (drug-target interactions hereafter). Drug molecules often interact not only with a therapeutic target but also with the other proteins (off-targets hereafter), which could lead to unwanted side effects [1]. Therefore, the identification of overall target proteins of drugs including the therapeutic targets and off-targets is a crucial process in the drug development. In addition, the understanding of the molecular mechanism of drug phenotypic effects in terms of drug-target interactions is also an important issue in many pharmaceutical applications. There is a hypothesis that drug phenotypic effects are involved in many kinds of biological features of drugs and proteins (e.g., drug chemical substructures, pharmacophores, protein functional sites, and biological pathways).

Recently, a variety of computational methods have been developed for large-scale prediction of drug-target interactions in the context of chemogenomics or pharmacogenomics. The key idea of the chemogenomic approach is that chemically similar compounds are likely to interact with similar proteins, and the prediction is performed based on compound chemical structures and/or protein sequences [2-7]. In contrast, the key idea of the pharmacogenomic approach is that phenotypically similar drugs are likely to interact with similar proteins, and the prediction is performed based on drug side effects and/or protein sequences [8-10]. However, the predictive models of most previous methods are not biologically interpretable, which makes it difficult to interpret biological features of drug-target interactions or compound-protein interactions.

The identification of biological features which are associated with drug-target interactions or compound-protein interactions is becoming a challenging issue in recent pharmaceutical science. In the context of chemogenomics, some machine learning methods with sparse models have been proposed to detect informative combinations of drug chemical substructures and protein domains which may explain the mechanism of drug-target interactions. The inferred features are called "chemogenomic features" [11,12]. In addition, the use of a data mining method has been proposed to infer molecular substructure pairs which appear frequently and significantly in interacting drug-target pairs [13]. The next challenge is to relate drug-target interactions with drug phenotypic effects (e.g., pharmaceutical effects and side effects). Recently, the use of drug targeted proteins has been proposed for predicting drug side effects [14-16]. The inference of proteins associated with drug side effects has been proposed [14,17], but there is no previous work on the analysis at the protein domain level. Protein domains are structural, evolutional, and functional units, so it would be important to investigate the associations between protein domains and drug side effects on a large scale.

In this paper, we make a systematic analysis of the correlation between drug side effects and protein domains, which we call "pharmacogenomic features," based on the drug-target interaction network. We detect drug side effects and protein domains that appear jointly and in known drug-target interactions, which is made possible by using classifiers with sparse models. It is shown that the inferred pharmacogenomic features can be used for predicting potential drug-target interactions. We also discuss advantages and limitations of the pharmacogenomic features, compared with the chemogenomic features that are the associations between drug chemical substructures and protein domains. To our knowledge, no other computational method has been reported for relating protein domains with drug side effects. The inferred side effect-domain association network is expected to be useful for estimating common drug side effects for different protein families and characteristic drug side effects for specific protein domains.

## Results and discussion

### Inference of pharmacogenomic features

We applied four methods: L1LOG, L2LOG, L1LOG-C, and L2LOG-C to infer pharmacogenomic features from the drug-target interaction network. Note that L1LOG and L2LOG are respectively *L*_1_- and *L*_2_-regularized logistic regressions with the tensor product descriptors, and L1LOG-C and L2LOG-C are respectively *L*_1_- and *L*_2_-regularized logistic regressions with the combined descriptors (see the Methods section for more details). In each method, we inferred pharmacogenomic features with positive weights in the predictive model.

**Figure 1:**
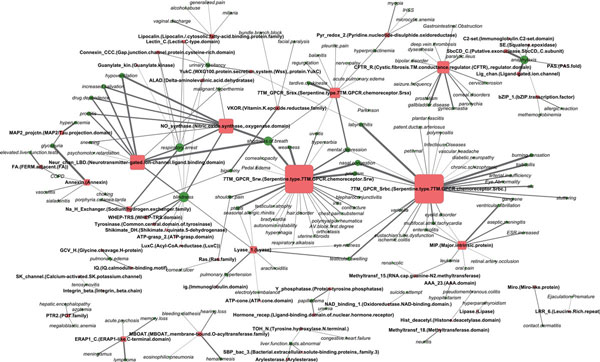
**Part of the inferred side effect and protein domain network**. Green circle and red rectangle represent a side effect and a protein domain, respectively. Node size represents a node degree. Edge width represents the weight of side effect and protein domain pair.

Each pharmacogenomic feature is composed of a side effect and a protein domain which are thought of as being associated with each other. There is a tendency that the protein domain in a pharmacogenomic feature are present in the same protein family targeted by drugs causing the side effect within the corresponding pharmacogenomic feature. We quantitated degree of the associations between side effects and protein domains by evaluating the weights on the corresponding pharmacogenomic features. Figure 1 shows a small part of side effect-domain association network inferred by the L1LOG method, where edges are placed between side effects and protein domains in positively weighted pharmacogenomic features and the top 200 weights are selected in the picture because of space limitation.

**Figure 2:**
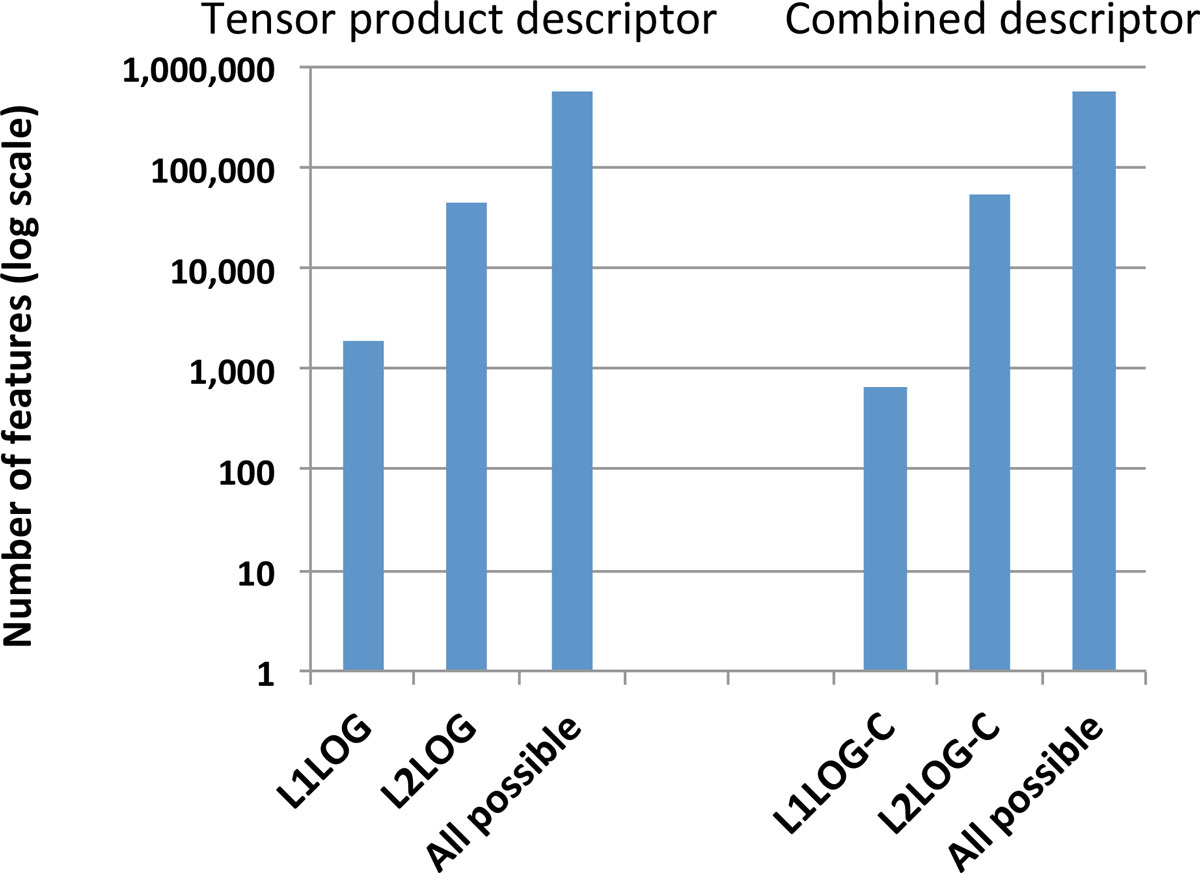
**Barplot of the numbers of features**. Comparison of the number of inferred features between L1LOG, L2LOG, L1LOG-C, and L2LOG-C.

We investigated the number of pharmacogenomic features inferred by the four methods. Figure 2 shows a summary of the comparison between L1LOG, L2LOG, L1LOG-C, and L2LOG-C on the gold standard data. It is found that the numbers of inferred features in L1LOG and L1LOG-C are significantly fewer than those in L2LOG and L2LOG-C, respectively. This observation means that the sparsity induced by the *L*_1 _penalty has positive effects of reducing the number of features in the descriptors. The feature extraction property helps us to analyze the inferred features for biological interpretation in practice.

**Figure 3:**
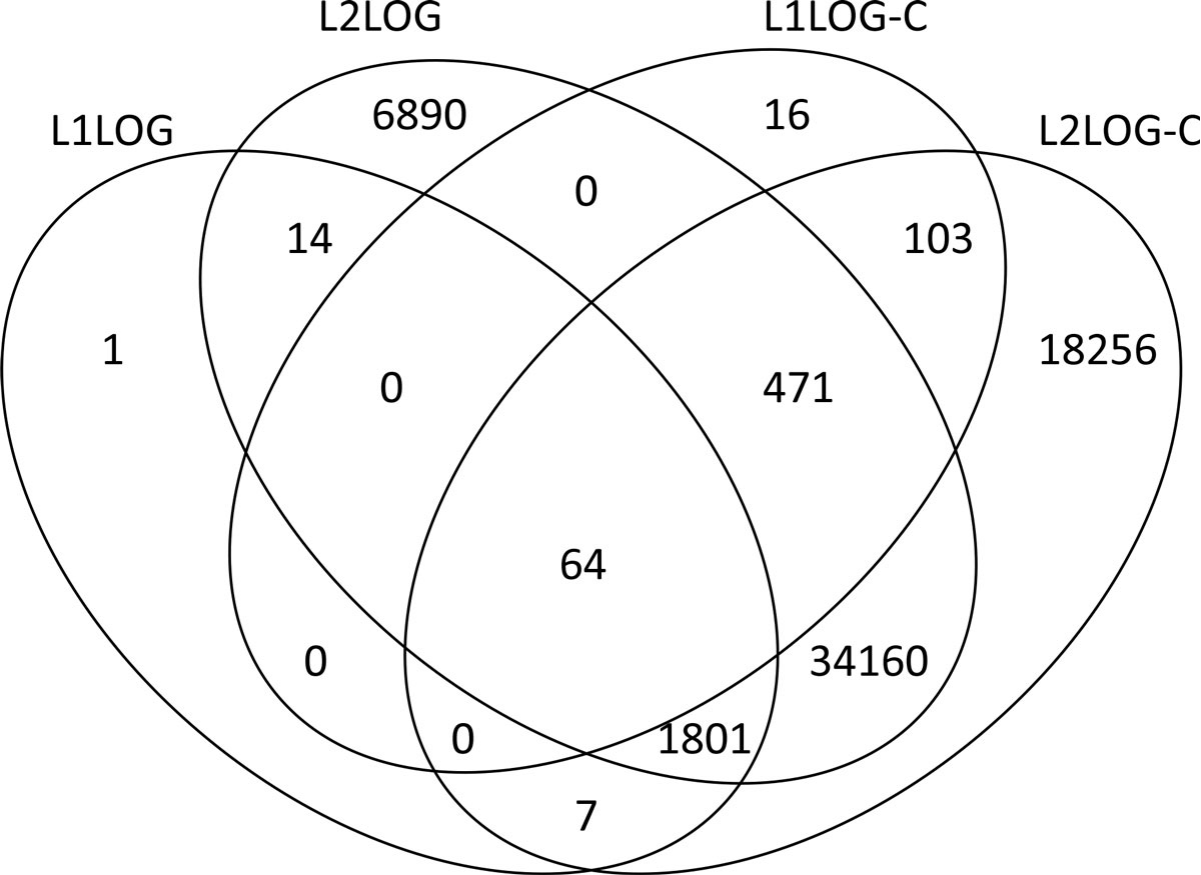
**Overlap of the number of features**. Comparison of the number of inferred features across L1LOG, L2LOG, L1LOG-C, and L2LOG-C.

Figure 3 shows the overlap of inferred pharmacogenomic features between the four methods. It is found that L1LOG was able to infer a very limited number of features and most of the features were included in those of L2LOG. This result suggests that the inferred features of L1LOG are more representative than those of L2LOG. Both L1LOG and L1LOG-C are sparsity-induced methods, but the number of common features between the two methods was very limited. This result suggests that biological interpretation about the inferred features may depend on the descriptors designed for drug-target pairs.

### Reconstruction of known drug-target interactions

We examined the validity of the pharmacogenomic features inferred by L1LOG, L2LOG, L1LOG-C, and L2LOG-C in terms of generalization properties for drug-target interactions. In order to test the ability of each method to reconstruct known drug-target interactions from the features, we performed two types of cross-validation experiments: pair-wise cross-validation and block-wise cross-validation (see the Methods section for more details). We also made a comparison between the pharmacogenomic features and the chemogenomic features. Note that the chemogenomic features correspond to the associations between drug chemical substructures and protein domains [12]. The methods with the pharmacogenomic features and the chemogenomic features are referred to as pharmacogenomic approach and chemogenomic approach, respectively, below.

We evaluated the performance by using the ROC curve (receiver operating characteristic curve). The ROC curve is a function of true positive rates against false positive rates based on many thresholds for the prediction scores, where true positives are correctly predicted interactions and false positives are incorrectly predicted interactions. We computed the total AUC score (the area under the ROC curve) over the five folds.

**Figure 4:**
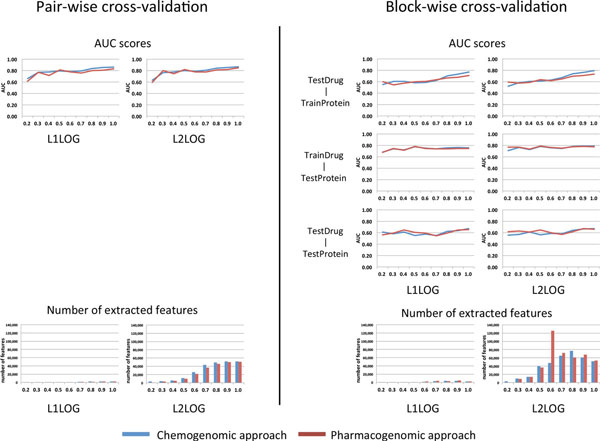
**AUC scores and the number of features in the cross-validation experiments by L1LOG and L2LOG**. The left panels show the results of the pair-wise cross-validation, while the right panels show the results of the block-wise cross-validation. The horizontal axis of each panel indicates the chemical structure similarity used for the clustering threshold in constructing the benchmark data.

Figure 4 shows the resulting AUC scores and the number of inferred features by L1LOG and L2LOG based on nine benchmark datasets with different clustering thresholds (see the Method section for more details). It is found that L1LOG is able to infer a smaller number of features, compared with L2LOG in most cases. Interestingly, the prediction accuracy of L1LOG is kept to some extent. Another observation is that the block-wise cross-validation provides lower AUC scores, compared with the pair-wise cross-validation. This result suggests that target prediction for new drugs with no target information and ligand prediction for orphan proteins with no ligand information are quite difficult problems.

Pharmacogenomic approach and chemogenomic approach showed similar behaviors in the pair-wise cross-validation setting, while the two approaches showed different behaviors in the block-wise cross-validation setting. The performance of pharmacogenomic approach was better than that of chemogenomic approach for the benchmark data consisting of structurally different drugs (i.e., in the case of low chemical similarity thresholds). On the other hand, the performance of pharmacogenomic approach was worse than the chemogenomic approach for benchmark data containing many structurally similar drugs (i.e., in the case of high chemical similarity thresholds). For example, the pharmacogenomic approach worked well for the TestDrug-TrainProtein pairs when the chemical similarity threshold is 0.2, and for the TestDrug-TestProtein pairs when the chemical similarity threshold lies in the range 0.2-0.5.

**Figure 5:**
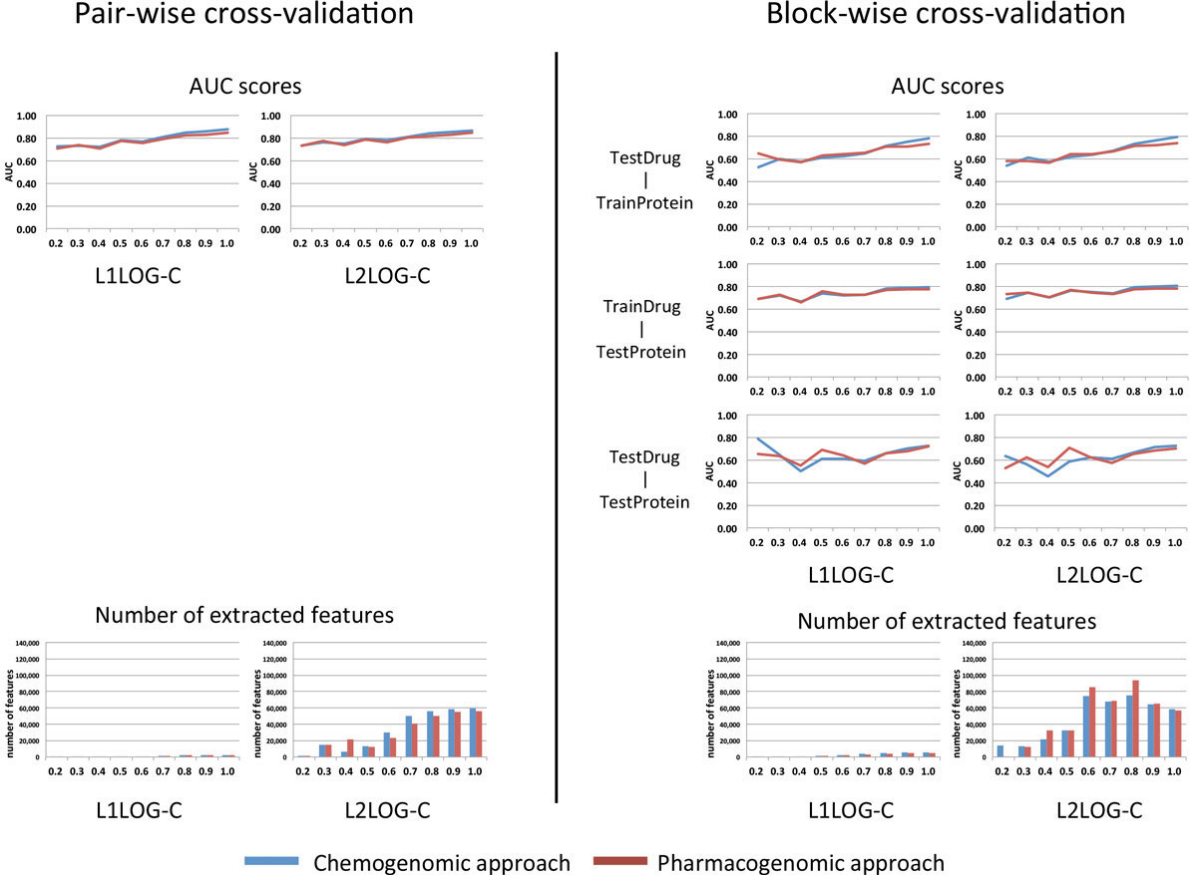
**AUC scores and the number of features in the cross-validation experiments by L1LOG-C and L2LOG-C**. The left panels show the results of the pair-wise cross-validation, while the right panels show the results of the block-wise cross-validation. The horizontal axis of each panel indicates the chemical structure similarity used for the clustering threshold in constructing the benchmark data.

Figure 5 shows the resulting AUC scores and the number of inferred features for L1LOG-C and L2LOG-C based on nine benchmark datasets. Note that L1LOG-C and L2LOG-C are based on the combination of the tensor product descriptor and individual feature vectors of drugs and target proteins. Similar tendencies exhibited in L1LOG and L2LOG can be observed in L1LOG-C and L2LOG-C as well. However, the AUC scores of L1LOG-C and L2LOG-C tend to be higher than those of L1LOG and L2LOG in both the pharmacogenomic approach and the chemogenomic approach. This result suggests that the combination of the tensor product descriptor and individual feature vectors of drugs and target proteins is meaningful for predicting drug-target interactions.

### Biological interpretation of side effects and protein domains in the inferred pharmacogenomic features

We made biological interpretations for drug side effects and protein domains in the inferred pharmacogenomic features. Table 1 shows examples of highly weighted side effect-domain pairs in the pharmacogenomic features inferred by the L1LOG method. Table 2 shows examples of highly weighted protein domains for each side effect inferred by the L1LOG method. Table 3 shows examples of highly weighted side effects for each protein domain inferred by the L1LOG method. The inferred side effect-domain associations suggest potential side effects involving each protein domain and side effects for a wide range of protein families. The results of all inferred pharmacogenomic features in Tables 1, 2, and 3 can be obtained from Additional files 1, 2, and 3 in supplemental materials. Here we discuss some examples in Tables 1, 2, and 3.

**Table 1:** Examples of highly weighted pharmacogenomic features inferred by the L1LOG method

Rank	Weight	Side effect	Protein domain ID	Protein domain definition
1	330.0000	anaphylaxis	PF13558	SbcCD_C (Putative exonuclease SbcCD, C subunit)
2	330.0000	tardive dyskinesia	PF10320	7TM_GPCR_Srsx (Serpentine type 7TM GPCR chemoreceptor Srsx)
3	330.0000	labyrinthitis	PF10316	7TM_GPCR_Srbc (Serpentine type 7TM GPCR chemoreceptor Srbc)
4	330.0000	shortness of breath	PF02898	NO_synthase (Nitric oxide synthase, oxygenase domain)
5	330.0000	priapism	PF10324	7TM_GPC_Srw (Serpentine type 7TM GPCR chemoreceptor Srw)
6	330.0000	nasal congestion	PF10324	7TM_GPCR_Srw (Serpentine type 7TM GPCR chemoreceptor Srw)
7	330.0000	nasal congestion	PF10316	7TM_GPCR_Srbc (Serpentine type 7TM GPCR chemoreceptor Srbc)
8	330.0000	weakness	PF10324	7TM_GPCR_Srw (Serpentine type 7TM GPCR chemoreceptor Srw)
9	327.1335	burning sensation	PF10316	7TM_GPCR_Srbc (Serpentine type 7TM GPCR chemoreceptor Srbc)
10	326.7361	glycosuria	PF00191	Annexin (Annexin)
11	325.3147	shortness of breath	PF10324	7TM_GPCR_Srw (Serpentine type 7TM GPCR chemoreceptor Srw)
12	324.4456	shortness of breath	PF02931	Neur_chan_LBD (Neurotransmitter-gated ion-channel ligand binding domain)
13	320.0000	anaphylaxis	PF00060	Lig_chan (Ligand-gated ion channel)
14	292.0382	hypoventilation	PF02898	NO_synthase (Nitric oxide synthase, oxygenase domain)
15	281.0425	hypoventilation	PF02931	Neur_chan_LBD (Neurotransmitter-gated ion-channel ligand binding domain)

**Table 2:** Examples of highly weighted protein domains for each side effect inferred by the L1LOG method

Rank	Weight	Side effect	Protein domain ID	Protein domain definition
1		anaphylaxis		
	330.0000		PF13558	SbcCD_C (Putative exonuclease SbcCD, C subunit)
	320.0000		PF00060	Lig_chan (Ligand-gated ion channel)
	115.8250		PF08491	SE (Squalene epoxidase)
	113.4802		PF00989	PAS (PAS fold)
	74.9419		PF05790	C2-set (Immunoglobulin C2-set domain)

2		tardive dyskinesia		
	330.0000		PF10320	7TM_GPCR_Srsx (Serpentine type 7TM GPCR chemoreceptor Srsx)

3		labyrinthitis		
	330.0000		PF10316	7TM_GPCR_Srbc (Serpentine type 7TM GPCR chemoreceptor Srbc)
	70.4820		PF10140	YukC (WXG100 protein secretion system (Wss), protein YukC)

4		shortness of breath		
	330.0000		PF02898	NO_synthase (Nitric oxide synthase, oxygenase domain)
	325.3147		PF10324	7TM_GPCR_Srw (Serpentine type 7TM GPCR chemoreceptor Srw)
	324.4456		PF02931	Neur_chan_LBD (Neurotransmitter-gated ion-channel ligand binding domain)
	100.7863		PF10320	7TM_GPCR_Srsx (Serpentine type 7TM GPCR chemoreceptor Srsx)

5		priapism		
	330.0000		PF10324	7TM_GPCR_Srw (Serpentine type 7TM GPCR chemoreceptor Srw)
	112.9922		PF10316	7TM_GPCR_Srbc (Serpentine type 7TM GPCR chemoreceptor Srbc)
	2.0630		PF00206	Lyase_1 (Lyase)

6		nasal congestion		
	330.0000		PF10324	7TM_GPCR_Srw (Serpentine type 7TM GPCR chemoreceptor Srw)
	330.0000		PF10316	7TM_GPCR_Srbc (Serpentine type 7TM GPCR chemoreceptor Srbc)

7		weakness		
	330.0000		PF10324	7TM_GPCR_Srw (Serpentine type 7TM GPCR chemoreceptor Srw)

8		burning sensation		
	327.1335		PF10316	7TM_GPCR_Srbc (Serpentine type 7TM GPCR chemoreceptor Srbc)
	14.2660		PF02867	Ribonuc_red_lgC (Ribonucleotide reductase, barrel domain)
	7.4168		PF03522	KCl_Cotrans_1 (K-Cl Co-transporter type 1 (KCC1))
	5.7520		PF10324	7TM_GPCR_Srw (Serpentine type 7TM GPCR chemoreceptor Srw)
	0.3741		PF00209	SNF (Sodium:neurotransmitter symporter family)

9		glycosuria		
	326.7361		PF00191	Annexin (Annexin)
	145.3436		PF08377	MAP2_projctn (MAP2/Tau projection domain)
	49.9279		PF03491	5HT_transporter (Serotonin (5-HT) neurotransmitter transporter, N-terminus)
	48.0767		PF02222	ATP-grasp (ATP-grasp domain)
	32.5342		PF00698	Acyl_transf_1 (Acyl transferase domain)

10		hypoventilation		
	292.0382		PF02898	NO_synthase (Nitric oxide synthase, oxygenase domain)
	281.0425		PF02931	Neur_chan_LBD (Neurotransmitter-gated ion-channel ligand binding domain)

**Table 3:** Examples of highly weighted side effects for each protein domain inferred by the L1LOG method

Rank	Weight	Protein domain ID	Protein domain definition	Side effect
1		PF13558	SbcCD_C (Putative exonuclease SbcCD, C subunit)	
	330.0000			anaphylaxis
	41.1178			allergic reaction

2		PF10320	7TM_GPCR_Srsx (Serpentine type 7TM GPCR chemoreceptor Srsx)	
	330.0000			tardive dyskinesia
	216.3340			mental depression
	102.3075			regurgitation
	100.7863			shortness of breath
	90.8773			hyperprolactinemia

3		PF10316	7TM_GPCR_Srbc (Serpentine type 7TM GPCR chemoreceptor Srbc)	
	330.0000			labyrinthitis
	330.0000			nasal congestion
	327.1335			burning sensation
	266.0200			torticollis
	225.7442			testicular swelling

4		PF02898	NO_synthase (Nitric oxide synthase, oxygenase domain)	
	330.0000			shortness of breath
	292.0382			hypoventilation
	232.9827			respiratory arrest
	229.9422			increased salivation
	204.4183			blindness

5		PF10324	7TM_GPCR_Srw (Serpentine type 7TM GPCR chemoreceptor Srw)	
	330.0000			priapism
	330.0000			nasal congestion
	330.0000			weakness
	325.3147			shortness of breath
	279.9735			ptosis

6		PF00191	Annexin (Annexin)	
	326.7361			glycosuria
	117.1047			vasculitis
	95.9706			sialadenitis
	79.3155			COPD
	71.9669			choking

7		PF02931	Neur_chan_LBD (Neurotransmitter-gated ion-channel ligand binding domain)	
	324.4456			shortness of breath
	281.0425			hypoventilation
	244.4901			increased salivation
	228.2349			respiratory arrest
	221.8014			drug dependence

8		PF00060	Lig-chan (Ligand-gated ion channel)	
	320.0000			anaphylaxis
	19.4911			allergic reaction

9		PF08377	MAP2_projctn (MAP2/Tau projection domain)	
	251.7744			hyperuricemia
	145.3436			glycosuria
	47.3304			sialadenitis
	44.6506			choking
	36.2648			polydipsia

10		PF14396	CFTR_R (Cystic fibrosis TM conductance regulator (CFTR), regulator domain)	
	239.2866			gallbladder disease
	212.0452			gynecomastia
	156.3952			paronychia
	134.2967			prostatism
	131.3925			cervical erosion

7TM_GPCR_Srw, 7TM_GPCR_Srbc, and 7TM_GPCR_Srsx are the Serpentine type 7TM GPCR chemoreceptors, which are the members of seven-transmembrane G-protein-coupled receptors (7TM GPCRs) that involved in many diseases and are also the target of many modern medicinal drugs. Srw, Srbc, and Srsx are the solo families amongst the superfamilies of chemoreceptors. It is reasonable to find that these three families share some side effects such as mental depression, nasal congestion, and priapism, however it might be meaningful to find that these families have their own specific side effects; shortness of breath, weakness, and ptosis for Srw, labyrinthitis, burning sensation and torticollis for Srbc, and tardive dyskinesia, hyperprolactinemia, and Parkinson for Srsx.

Neur_chan_LBD (Neurotransmitter-gated ion-channel ligand binding domain) is a transmembrane receptor-ion channel complex that binds specific ligands for rapid transmission of signals at chemical synapses, which includes nicotinic acetylcholine receptor (AchR), glycine receptor, gamma-aminobutyric acid (GABA) receptor, serotonin 5HT3 receptor, and glutamate receptor. By viewing the side effects on the protein domain level, this domain was shown to be involved in many side effects, such as shortness of breath, respiratory arrest, blindness, hypoventilation, increased salivation, drug dependence, and proctitis. It is understandable that most of these side-effects are shared by NO_synthase (Nitric oxide synthase, oxygenase domain). NO_synthase has isoenzymes eNOS (endothelial NOS) and nNOS (neuronal NOS); the former is the primary signal generator in the control of vascular tone, insulin secretion, and airway tone, and the latter is involved in the development of nervous system.

### Novel predictions

Finally, we conducted a large-scale prediction of unknown interactions of all drugs and all proteins based on the pharmacogenomic features inferred by L1LOG. We learned a predictive model based on all drug-target pairs in the gold standard data, and applied it to all drugs and proteins for which side-effect information and domain information are available. We put the list of the top 1000 predictions in Additional file 4.

## Conclusion

In this paper we made a systematic analysis of the correlation between drug side effects and protein domains, which we call pharmacogenomic features, using binary classifiers with sparse models based on the drug-target interaction network. We showed the usefulness of the inferred pharmacogenomic features for predicting drug-target interactions. To our knowledge, this work is the first study to relate protein domains with drug side effects on a large scale.

In this study, we used logistic regression as a binary classifier, but other classifiers can be used for the same objective. For example, support vector machine (SVM) is a good candidate for high-performance binary classifier. Actually, we performed the same analysis using SVM in a similar manner as logistic regression, and confirmed that the same tendency in the results can be obtained. The detailed results can be found in Additional files 5 and 6.

In this study we used side effect profiles of drugs and domain profiles of target proteins in the correlation analysis, but the performance and the biological interpretation depend heavily on the elements in the profiles of drugs and proteins. The method can not extract features which are absent from the predefined descriptors, so the generalization properties of the method could be improved by constructing more meaningful descriptors or using more complete descriptors.

## Materials and methods

### Data

#### Drug-target interactions

We obtained the information about drug-target interactions from the DrugBank database [18]. The number of interactions in the dataset is 1064. These interactions involve 413 drugs and 400 target proteins. We used this data set as gold standard data in the cross-validation experiment.

#### Pharmacological and chemical data of drugs

We obtained the information about side effects of drugs from the SIDER database that accumulates reported side effects from package inserts for marketed drugs [19]. We represented each drug by a 1179-dimensional binary vector in which the presence or absence of each side effect is coded as 1 or 0.

We obtained the information about chemical structures of drugs from the PubChem database [20]. We represented each drug by an 881-dimensional binary vector in which 881 PubChem substructures are used and the presence or absence of each substructure is coded as 1 or 0.

#### Genomic and functional data of target proteins

We obtained genomic information about target proteins from the UniProt database [21], and obtained the protein domains from the PFAM database [22]. We represented each target protein by a 476-dimensional binary vector in which 476 PFAM domains are used and the presence or absence of each domain is coded as 1 or 0.

### Classifiers for drug-target pairs

We consider the feature extraction problem in the context of drug-target interaction prediction. We represent a pair of drug *D *and protein *P *by (*D*, *P*). Suppose that we are given a learning set of drug-target pairs (*D_i_*, *P_j_*) (*i *= 1, 2, . . . , *n_D_*; *j *= 1, 2, . . . , *n_P_*), where the pairs are known to interact or not, *n_D _*is the number of drugs and *n_P _*is the number of target proteins in the learning set.

We represent a pair of drug *D *and protein *P *by a feature vector Φ(*D*, *P*), and then estimate a function *f *(*D*, *P*) = ***w***^T^Φ(*D*, *P*) which would predict whether drug-target pair (*D*, *P*) is an interacting pair or not. We optimize the weight vector ***w ***based on the learning set with label information.

The feature vector of drug *D *is supposed to be represented as an *M*-dimensional binary vector:

$$\text{Φ}\left(D\right)={\left(d_{\text{1}},d_{\text{2}},...,d_{M}\right)}^{\text{T}},$$

where *d_k _*∈ {0, 1}, *k *= 1, . . . , *M *. For example, Φ(*D*) is a profile of side effects or chemical substructures in this study. In the same manner, the feature vector of protein *P *is supposed to be represented as an *N*-dimensional binary vector:

$$\text{Φ}\left(P\right)={\left(p_{\text{1}},p_{\text{2}},...,p_{N}\right)}^{\text{T}},$$

where *p_l _*∈ {0, 1}, *l *= 1, . . . , *N *. For example, Φ(*P*) is a profile of protein domains in this study.

We propose two kinds of feature vectors for each drug-target pair. First, we represent each drug-target pair by the tensor product between Φ(*D*) and Φ(*P*) as follows:

$$\begin{array}{c} \text{Φ}\left(D,P\right)=\text{Φ}\left(D\right)⊗\text{Φ}\left(P\right)\\ \;\;\;\;\;\;\;\;\;\;\;\;\;\;\;\;\;\;\;\;\;\;\;\;={\left(d_{\text{1}}p_{\text{1}},...,d_{\text{1}}p_{N},...,d_{M}\;p_{\text{1}},...,d_{M}p_{N}\right)}^{\text{T}}, \end{array}$$

where Φ(*D*, *P *) is an (*M *× *N*)-dimensional feature vector. We refer to the feature vector as "tensor product descriptor" in this study. This tensor product descriptor is similar to that in the previous work [12].

Second, we represent each drug-target pair by the combination of the tensor product descriptor Φ(*D*) ⊗ Φ(*P *) and individual feature vectors Φ(*D*) and Φ(*P*) as follows:

$$\begin{array}{c} {\text{Φ}}_{C}\left(D,P\right)={\left[{\left(\text{Φ}\left(D\right)⊗\text{Φ}\left(P\right)\right)}^{\text{T}},\text{Φ}{\left(D\right)}^{\text{T}},\text{Φ}{\left(P\right)}^{\text{T}}\right]}^{\text{T}}\\ \;\;\;\;\;\;\;\;\;\;\;\;\;\;\;\;\;\;\;\;\;\;\;\;\;\;\;={\left(d_{\text{1}}p_{\text{1}},...,d_{\text{1}}p_{N},...,d_{M}p_{\text{1}},...,d_{M}p_{N},d_{\text{1}},d_{\text{2}},...,d_{M},p_{\text{1}},p_{\text{2}},...,p_{N}\right)}^{\text{T}}, \end{array}$$

where Φ*_C _*(*D*, *P*) is an (*M *× *N *+ *M *+ *N*)-dimensional binary vector. We refer to the feature vector as "combined descriptor" in this study.

In this study we use logistic regression as a binary classifier to predict whether a drug *D *interacts with a target protein *P *or not. The predictive model is usually learnt by minimizing the loss function with *L*_2_-regularization. However, *L*_2_-regularization tends to keep most weight elements to be non-zeros, which makes it difficult to interpret features from the resulting weight vector. Another possible solution is to use *L*_1_-regularization that tends to make most weight elements to be zeros, which makes it easier to interpret features from the resulting weight vector. Therefore, we introduce a logistic regression model with *L*_1_-regularization.

Suppose that we have a learning set of drug-target pairs and interaction labels

(Φ(*D_i_*, *P_j_*), *y_ij_*), *y_ij _*∈ {+1, *−*1} (*i *= 1, 2, . . . , *n_D_*; *j *= 1, 2, . . . , *n_P_*), where *n_D _*is the number of drugs and *n_P _*is the number of target proteins in the learning set. The weight vector ***w ***of the linear logistic regression is usually learned with *L*_2_-regularization as follows:

$$\underset{w}{\text{min}||}w||{}_{2}+C\overset{n_{D}}{\underset{i=1}{\sum }}\overset{n_{P}}{\underset{j=1}{\sum }}\text{log}\left(\text{1}+\text{exp}\left(-y_{ij}\;w^{\text{T}}\text{Φ}\left(D_{i},P_{j}\right)\right)\right),$$

where *|| *· *||*_2 _is *L*_2 _norm (the sum of squared values) and *C *is a regularization parameter to control the penalty.

To induce sparsity in the model, the weight vector ***w ***of the linear logistic regression is learned with *L*_1_-regularization as follows:

$$\underset{w}{\text{min}||}w||{}_{1}+C\overset{n_{D}}{\underset{i=1}{\sum }}\overset{n_{P}}{\underset{j=1}{\sum }}\text{log}\left(\text{1}+\text{exp}\left(-y_{ij}\;w^{\text{T}}\text{Φ}\left(D_{i},P_{j}\right)\right)\right),$$

where *|| *· *||*_1 _is *L*_1 _norm (the sum of absolute values) and *C *is a regularization parameter to control the sparsity. We examine various values (0.0001, 0.001, 0.01, 0.1, 1, 10, 100, 1000, 10000) for the hyper parameter *C*, and select the value that gave the highest AUC score in the cross-validation experiment.

In practice, we consider applying the logistic regression with the tensor product descriptor Φ(*D*, *P*) and with the combined descriptor Φ*_C_*(*D*, *P*). We refer to *L*_1_-regularized logistic regression with the tensor product descriptor as "L1LOG," and *L*_2_-regularized logistic regression with the tensor product descriptor as "L2LOG," respectively. We refer to *L*_1_-regularized logistic regression with the combined descriptor as "L1LOG-C," and *L*_2_-regularized logistic regression with the combined descriptor as "L2LOG-C," respectively.

### Cross-validation experiments for benchmark data

There are two scenarios for drug-target interaction prediction from practical viewpoints. The first scenario is that we have drugs with target information and proteins with ligand information, and the goal is to additionally detect missing interactions between the drugs and the proteins. The second scenario is that we have drugs with no target information and protein with no ligand information, and the goal is to find all potential target proteins of the drugs and all potential ligands of target proteins. To simulate the above two scenarios in the 5-fold cross-validation experiment, we consider two different settings: pair-wise cross-validation and block-wise cross-validation.

The pair-wise cross-validation consists of the following procedures: First, we split all drug-target pairs in the gold standard set into five subsets of all drug-target pairs in an independent manner. Second, we regard each subset of drug-target pairs as a test set, and regard the other four subsets of drug-target pairs as a training set. Third, we optimize a predictive model based on drug-target pairs in the training set. Finally, we apply the predictive model to drug-target pairs in the test set. Note that drug-target pairs are considered independent of each other, so drugs and target proteins in test pairs are overlapped with those in the training set to some extent.

The block-wise cross-validation consists of the following procedures: First, we split drugs and target proteins in the gold standard set into five subsets of drugs and five subsets of target proteins. Second, we regard each subset of drugs (resp. proteins) as test drugs (resp. test proteins), and use the other four subsets of drugs as training drugs (resp. training proteins). Third, we optimize a predictive model based on drug-target pairs consisting of training drugs and training proteins. Finally, we compute the prediction scores for three types of drug-target pairs: test drugs v.s. training target proteins (referred to as "TestDrug-TrainProtein"), training drugs v.s. test target proteins (referred to as "TrainDrug-TestProtein"), and test drugs v.s. test target proteins (referred to as "TestDrug-TestProtein"). Note that drugs and proteins in test pairs are not completely different from those in the training set. Thus, the prediction problem in the block-wise setting is more difficult than that in the pair-wise setting.

The gold standard data contain many drugs which were chemically and structurally almost identical, because they were derived from the same lead compound. If these identical drugs were divided into a training set and a test set, the prediction in the cross-validation experiment would be trivial. To avoid overestimation of the prediction accuracy, we perform a grouping of similar drugs based on their chemical structures and use only drugs which are chemically and structurally different to some extent, following a previous work [10]. First, we carry out a clustering of all drugs based on Tanimoto coefficient (Jaccard coefficient) [23] of chemical fingerprints using average linkage algorithm. Second, we cluster drugs with high Tanimoto coefficients into the same cluster, and selected one representative drug within each cluster. Third, we construct a set of drugs with low Tanimoto coefficients. Finally, we prepare nine sets of benchmark data consisting of representative drugs by varying the clustering threshold little by little (e.g., from 0.2 to 1.0 by 0.1) on the dendrogram. When the clustering threshold is 0.1, the number of drug clusters is only 3 in our data, so it is not possible to test the clustering threshold of 0.1 in the 5-fold cross-validation.

## Competing interests

The authors declare that they have no competing interests.

## Authors' contributions

HI tested the performance of the methods and drafted the manuscript. SM prepared the datasets and made biological interpretations of the results. YT implemented the algorithm of the methods. MK made biological interpretations of the results and drafted the manuscript. SG drafted the manuscript. YY directed the work, and drafted the manuscript. All authors read and approved the final manuscript.

## Supplementary Material

Additional file 1 **Extracted side effects and protein domains of L1LOG**. Click here for file

Additional file 2 **Extracted protein domains for each side effect of L1LOG**. Click here for file

Additional file 3 **Extracted side effects for each protein domain of L1LOG**. Click here for file

Additional file 4 **The list of novel drug-target predictions of L1LOG**. Click here for file

Additional file 5 **AUC scores and the number of features in the pair-wise and block-wise cross-validation experiments by L1LOG, L2LOG, L1SVM, and L2SVM**. Click here for file

Additional file 6 **AUC scores and the number of features in the pair-wise and block-wise cross-validation experiments by L1LOG-C, L2LOG-C, L1SVM-C, and L2SVM-C**. Click here for file

## Declarations

The publication cost for this work was supported by JSPS Kakenhi 25700029.

This article has been published as part of *BMC Systems Biology *Volume 7 Supplement 6, 2013: Selected articles from the 24th International Conference on Genome Informatics (GIW2013). The full contents of the supplement are available online at http://www.biomedcentral.com/bmcsystbiol/supplements/7/S6.
