# Supplementary figures and images for: Inferring protein domains associated with drug side effects based on drug-target interaction network
Source: BMC Syst Biol. 2013 Dec 13;7(Suppl 6):S18. doi: 10.1186/1752-0509-7-S6-S18 (PMC4029543; doi:10.1186/1752-0509-7-S6-S18)

## Pair-wise cross-validation

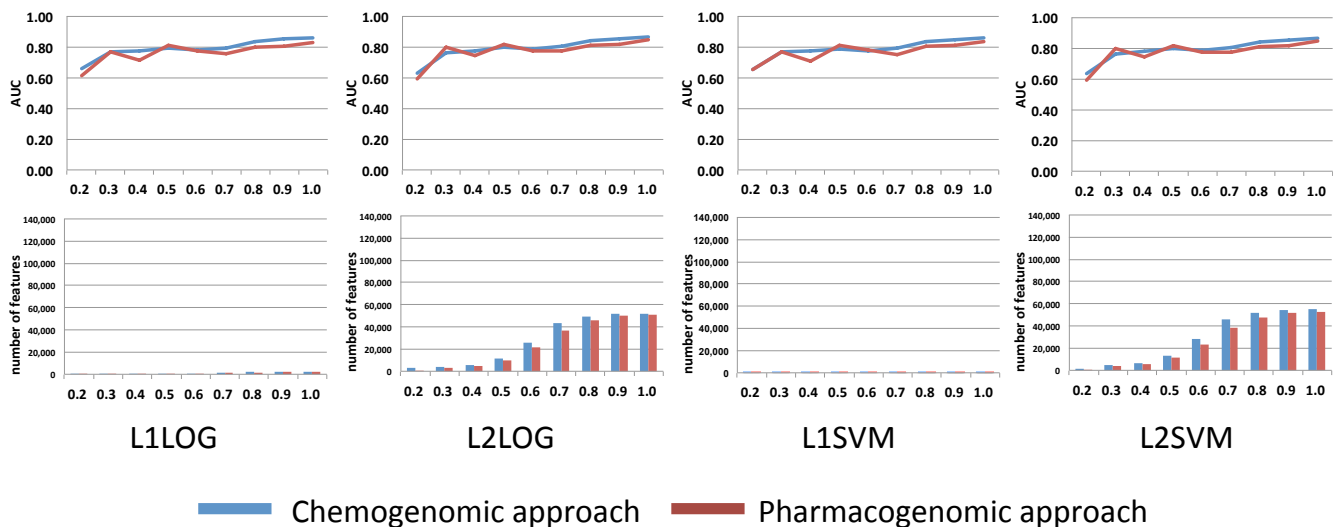

## Block-wise cross-validation

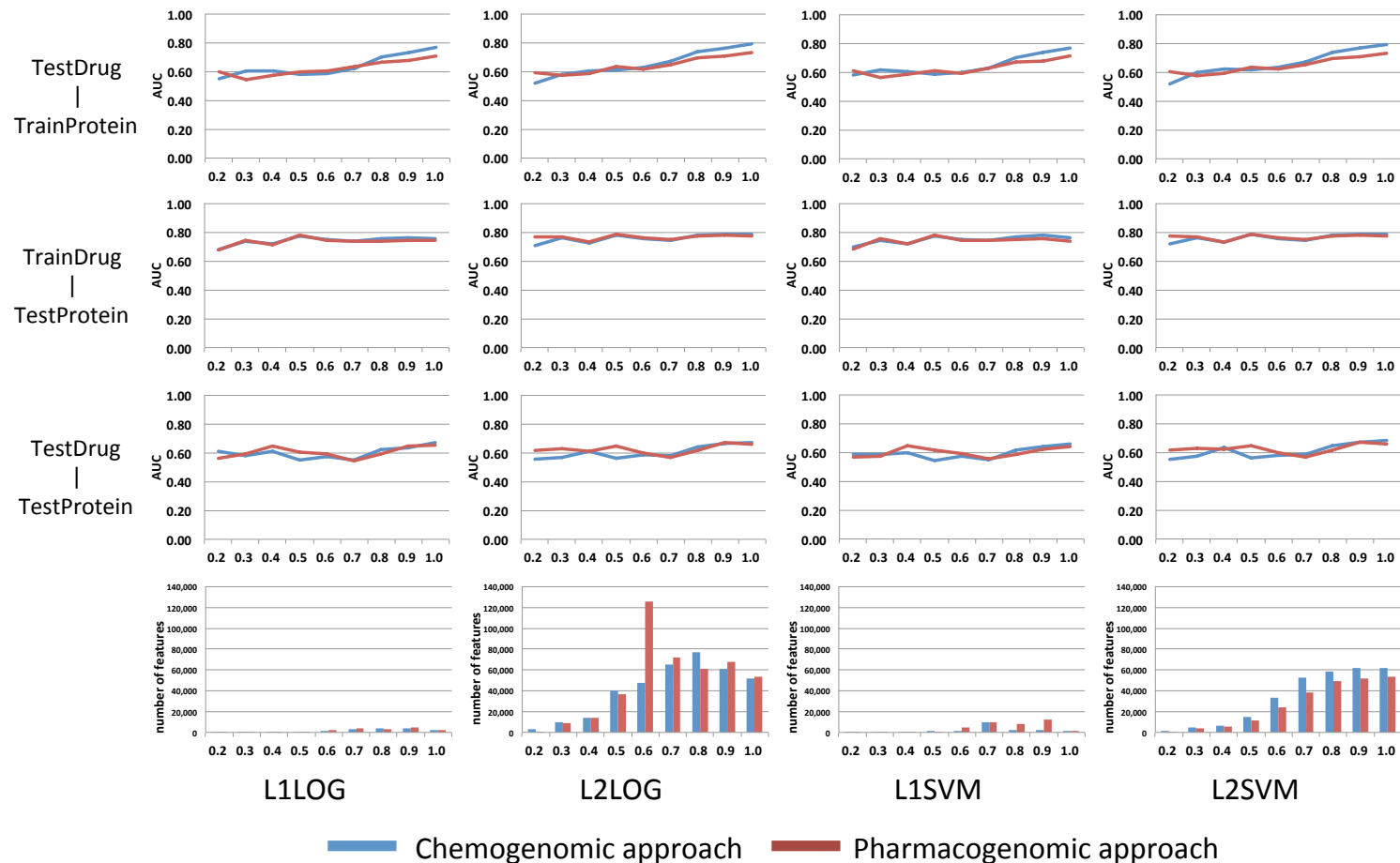

Supplement: Additional file 5 — AUC scores and the number of features in the pair-wise and block-wise cross-validation experiments by L1LOG, L2LOG, L1SVM, and L2SVM. [file 1752-0509-7-S6-S18-S5.PDF]

## Pair-wise cross-validation

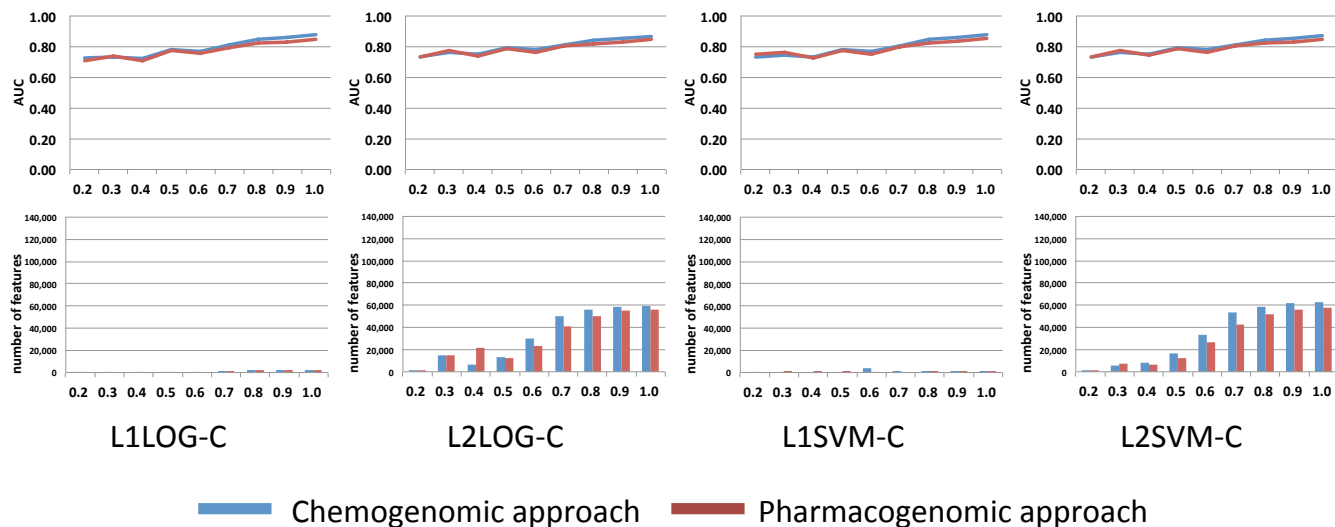

## Block-wise cross-validation

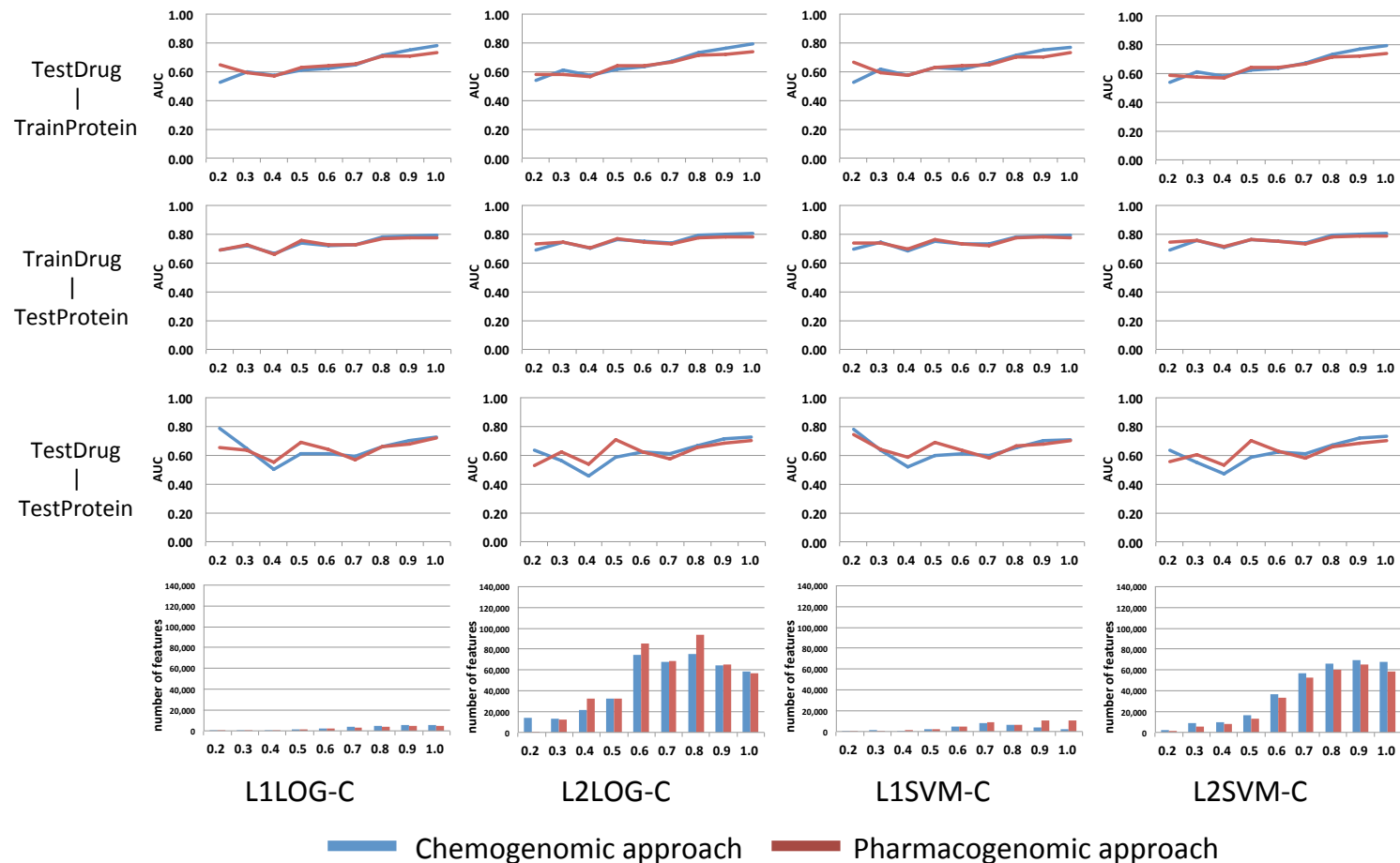

Supplement: Additional file 6 — AUC scores and the number of features in the pair-wise and block-wise cross-validation experiments by L1LOG-C, L2LOG-C, L1SVM-C, and L2SVM-C. [file 1752-0509-7-S6-S18-S6.PDF]
